# Isolated bladder metastasis from lung adenocarcinoma: a case report

**DOI:** 10.1093/jscr/rjab195

**Published:** 2021-05-27

**Authors:** Selim Zaghbib, Marouene Chakroun, Ahmed Saadi, Hamza Boussaffa, Nadia Znaidi, Soumaya Rammeh, Haroun Ayed, Mohamed Chebil

**Affiliations:** Department of Urology, Charles Nicolle Hospital, Tunis, Tunisia; Department of Urology, Charles Nicolle Hospital, Tunis, Tunisia; Department of Urology, Charles Nicolle Hospital, Tunis, Tunisia; Department of Urology, Charles Nicolle Hospital, Tunis, Tunisia; Department of Pathology, Charles Nicolle Hospital, Tunis, Tunisia; Department of Pathology, Charles Nicolle Hospital, Tunis, Tunisia; Department of Urology, Charles Nicolle Hospital, Tunis, Tunisia; Department of Urology, Charles Nicolle Hospital, Tunis, Tunisia

## Abstract

Cancers of extra bladder origin represent between 2 and 12% of bladder neoplasms and are most often secondary to contiguous bladder invasion. Metastasis from distant organs is exceptional, especially from pulmonary adenocarcinoma with <10 cases identified over the last 20 years. We report here a new case of a 55-year-old patient with a recently diagnosed pulmonary adenocarcinoma referred to the urology department for macroscopic hematuria. Computed tomography scan showed, in addition to the lung mass of the right lower lobe with a right mediastinal adenopathy, a thickening of the right lateral bladder wall. Cystoscopy showed inflammatory lesions on the bladder mucosa, which biopsy with immunohistochemical examination revealed to be tumoral proliferation in the lamina propria realizing the same immunohistochemical profile of the primary lung tumor (CK7+/TTF1+/CK20−/PSA−). The patient was treated with palliative platinum-based chemotherapy and unfortunately died 5 months after diagnosis.

## INTRODUCTION

Metastatic spread to the bladder from distant cancer is very rare. Indeed, cancers of extra bladder origin represent between 2 and 12% of bladder neoplasms and are most often secondary to invasion of the bladder by contiguity [1, 2]. Metastasis from distant organs is exceptional, especially from lung adenocarcinoma with <10 cases identified in the last 20 years [3]. This article reports a new case of a bladder metastasis from lung adenocarcinoma, revealed by macroscopic hematuria.

Our work has been reported in line with the CARE criteria [4].

## CASE PRESENTATION

**Figure 1:**
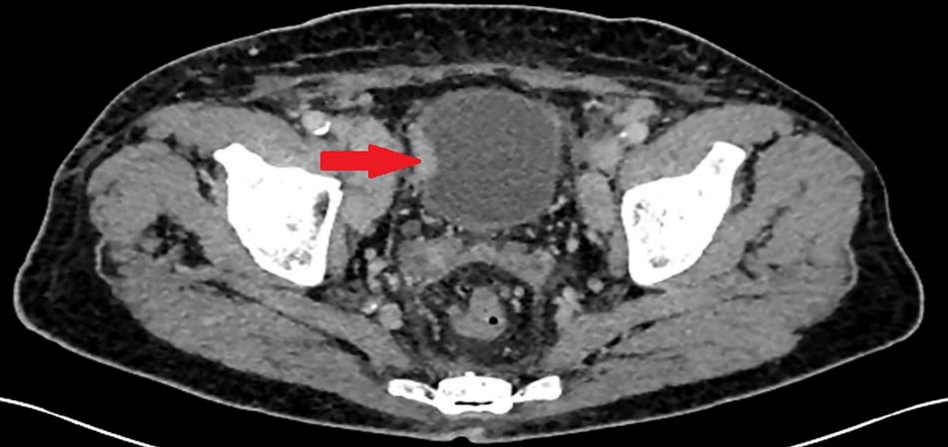
Thickening of the right lateral wall of the bladder enhancing at arterial time on CT scan (arrow).

**Figure 2:**
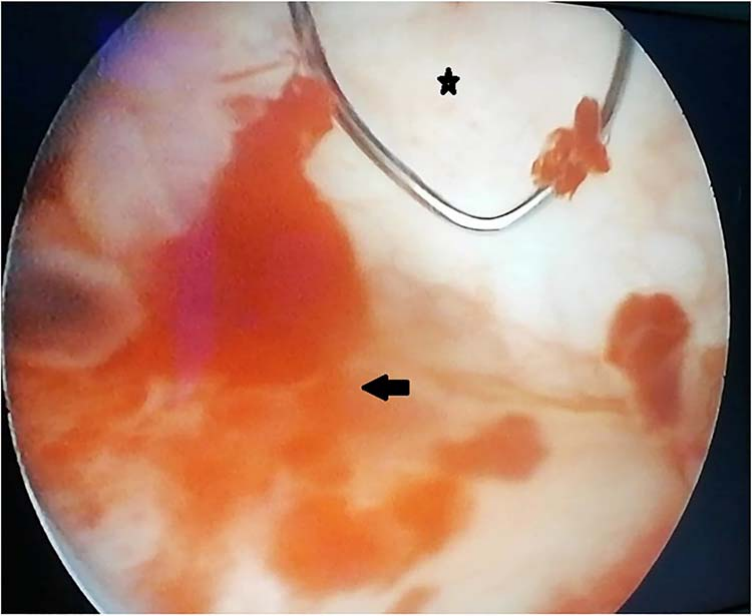
Peroperative appearance during cystoscopy before resection. Star: healthy bladder mucosa. Arrow: bullous inflammatory lesion that was resected.

A 55-year-old, nonsmoking patient with no medical or surgical history who had a pulmonary adenocarcinoma diagnosed 1 month prior was referred to the urology department for macroscopic hematuria. Actually, the patient had initially presented with hemoptysis and a computed tomography (CT) scan revealed a right lower lobar pulmonary nodule, which biopsies concluded to a moderately differentiated adenocarcinoma. Interrogation revealed urinary frequency, urgency and several episodes of intermittent hematuria for 3 months. Physical examination showed a patient with an impaired general condition, without other abnormalities and a normal urine color. The urine dipstick revealed a hematuria at two crosses. The biological workup showed no abnormalities and the prostate specific antigen (PSA) level was at 2 ng/ml. The thoracic-abdominal-pelvic CT scan, performed for his lung cancer, showed, in addition to the lung mass of the right lower lobe with a right mediastinal adenopathy, a thickening of the bladder wall enhancing on the right lateral wall, an upper urinary tract free of any lesion and no distant metastases (Fig. 1). Cystoscopy under locoregional anesthesia was performed, showing a normal endoscopic appearance of the urethra and prostate gland, with inflammatory and bullous lesions on the bladder trigone and right lateral wall, which were biopsied using a resector (Fig. 2).

**Figure 3:**
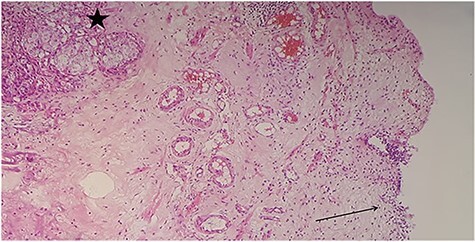
Urothelial mucosa (arrow) with carcinomatous proliferation (star) composed of nodules within a sparse fibrous stroma (hematoxylin and eosin [HE] ×100).

Anatomopathological examination of the bladder biopsies showed a carcinomatous proliferation in the lamina propria, made of globular cells with abundant eosinophilic cytoplasm and voluminous nucleoli, forming nodules within a sparse fibrous stroma (Fig. 3).

**Figure 4:**
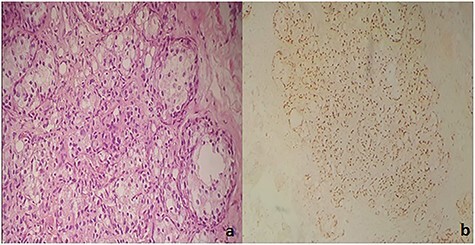
(**a**) Carcinomatous proliferation consisting of globular cells with abundant eosinophilic cytoplasm and a large nucleolus (HE ×250). (**b**) Diffuse nuclear staining of tumor cells by anti-TTF1.

Immunohistochemical examination showed diffuse nucleolar staining of the tumor cells by CK7 and anti-TTF1 and absence of staining by CK20 and PSA thus realizing the same immunohistochemical profile of the primary lung tumor (Fig. 4).

The patient was treated with palliative platinum-based chemotherapy and died 5 months after diagnosis.

## DISCUSSION

Bladder cancer is one of the most common genitourinary cancers and is represented by urothelial (transitional cell) carcinoma in >95% of cases; metastases from a distant primary tumor represent <2% of bladder cancers [5]. Indeed, the bladder represents the second most common genitourinary metastatic location after the kidney [6]. Primary tumor locations with bladder metastases described in the literature are (from most to least common): stomach, melanoma, breast and lung. According to a large autopsy study, bladder metastasis was observed in 0.16% of metastatic lung cancers [7]. Only 11 cases have been reported in the literature, 8 of which were from adenocarcinoma [3].

Clinically, the circumstance of diagnosis of bladder metastasis was macroscopic hematuria in most of the described cases [3]. However, this is a delayed symptom, as the metastases are initially located in the lamina propria, and the hematuria is indicative of urothelial involvement and ulceration [8]. Other modes of presentation include hydronephrosis, pelvic pain and dysuria [3].

CT scan, usually done as part of staging or prompted by hematuria, may show thickening or thinning of the bladder wall. However, it may be normal, as in the case described [3].

Cystoscopy shows variable, nonspecific appearances and may even suggest a primary bladder tumor [9]. However, the most frequently described appearance is a single lesion, in contrast to our case [3]. Since tumor proliferation starts deeper to the mucosa, cystoscopy may be normal, especially in the absence of hematuria [8].

The positive diagnosis is based on pathological examination of the bladder resection specimens. The main differential diagnosis is primary adenocarcinoma of the bladder. Certain morphologic criteria are in favor of the primary nature: associated intestinal metaplasia, glandular cystitis, *in situ* adenocarcinoma, urothelial carcinomatous transformation [10]. Arguments in favor of the secondary character are: deep location in the mucosa, integrity of the urothelium, absence of *in situ* lesions, diffuse lymphovascular invasion [11]. However, although suggestive, the morphological study is not sufficient. It is the clinical context, combined with an immunohistochemical study that allows the diagnosis to be made. The combination of CK7/CK20 markers allows orientating toward the origin of the adenocarcinoma [12]. The CK7+/CK20+ or CK20-suggested phenotype suggests a pulmonary or bladder origin. The TTF-1 marker, expressed by primary lung adenocarcinoma, has high sensitivity and specificity for the diagnosis of lung adenocarcinoma metastatic to the bladder.

Treatment is similar to that of metastatic lung adenocarcinoma, which is based on targeted therapy or chemotherapy depending on the presence of the EGFR or ALK mutation and on symptomatology [13].

## CONCLUSION

Bladder metastases from lung adenocarcinoma are extremely rare with <10 cases described in the literature. Their diagnosis is delicate and poses the problem of differential diagnosis with a primary bladder tumor or another nonpulmonary primary and is based on immunohistochemical criteria. The prognosis is similar to that of metastatic pulmonary adenocarcinoma.

## CONSENT FOR PUBLICATION

Written informed consent was obtained from the patient for publication of this case report and accompanying images.

## CONFLICT OF INTEREST STATEMENT

None declared.

## FUNDING

None.

## References

[1] Melicow MM . Tumours of the urinary bladder: a clinicopathological analysis of over 2500 specimens and biopsies. J Urol 1955;74:498–521. 13264313 10.1016/S0022-5347(17)67309-9

[2] Bates AW, Baithun SI. Secondary neoplasms of the bladder are histological mimics of nontransitional cell primary tumours: clinicopathological and histological features of 282 cases. Histopathology 2000;63:32–40. 10.1046/j.1365-2559.2000.00797.x 10632749

[3] Sanguedolce F, Loizzi D, Sollitto F, Di Bisceglie M, Lucarelli G, Carrieri G, et al. Bladder metastases from lung cancer: clinical and pathological implications: a systematic review. Oncology 2017;92:125–34. 28056456 10.1159/000454731

[4] Gagnier JJ, Kienle G, Altman DG, et al. The CARE guidelines: consensus-based clinical case reporting guideline development. Glob Adv Health Med 2013;2:38–43. 10.7453/gahmj.2013.008 PMC3833570 24416692

[5] Mizutani Y, Hashimura T, Kitayama T, Toshimitsu T, Nonomura M. A case of secondary tumor the origin (gastric cancer) of which could not be identified before autopsy. Hinyokika Kiyo 1990;36:605–8. 2169186

[6] Bivalacqua TJ, Alphs H, Schaeffer EM, Schoenberg MP, Aksentijevich I. Paraneoplastic polyarthritis from non-small-cell lung cancer metastatic to the bladder. J Clin Oncol 2007;25:2621–3. 17577044 10.1200/JCO.2007.11.5600

[7] Disibio G, French SW. Metastatic patterns of cancers: results from a large autopsy study. Arch Pathol Lab Med 2008;132:931–9. 18517275 10.5858/2008-132-931-MPOCRF

[8] Lan SK, Lin YH, Tzai TS, Tsai YS. A rare instance of lung cancer metastasizing to the urinary bladder. J Taiwan Urol Assoc 2006;17:63–6

[9] Cormio L, Sanguedolce F, Di Fino G, Massenio P, Liuzzi G, Bufo P, et al. Bladder metastasis from lung adenocarcinoma: a difficult differential diagnosis with primary bladder adenocarcinoma. World J Surg Oncol 2014;12:90. 24716732 10.1186/1477-7819-12-90 PMC3984282

[10] Abrams HL, Spiro R, Goldstein N. Metastases in carcinoma; analysis of 1,000 autopsied cases. Cancer 1950;3:74–85. 15405683 10.1002/1097-0142(1950)3:1<74::aid-cncr2820030111>3.0.co;2-7

[11] Haupt HM, Mann RB, Trump DL, Abeloff MD. Metastatic carcinoma involving the testis. Clinical and pathologic distinction from primary testicular neoplasms. Cancer 1984;10:592–5. 10.1002/1097-0142(1984)54:4<709::aid-cncr2820540419>3.0.co;2-6 6204734

[12] Lin F, Liu H. Immunohistochemistry in undifferentiated neoplasm/tumor of uncertain origin. Arch Pathol Lab Med 2014;138:1583–610. 25427040 10.5858/arpa.2014-0061-RA

[13] Reck M, Popas S, Reinmuth N, et al. Metastatic non-small-cell lung cancer (NSCLC): ESMO Clinical Practice Guidelines for diagnosis, treatment and follow-up. Ann Oncol 2014;25:S27–39. 10.1093/annonc/mdu199 25115305

